# Follow-up intensity after colorectal cancer surgery in patients aged ≤ 50, 50–70 and > 70 years – an analysis within the COLOFOL randomised clinical trial

**DOI:** 10.1007/s00384-026-05096-9

**Published:** 2026-01-26

**Authors:** Ida Gutlic, Katalin Veres, Erzsébet Horváth-Puhó, Marie-Louise Lydrup, Pamela Buchwald

**Affiliations:** 1 Department of Clinical Sciences Malmö, Lund University, 205 02 Malmö, Sweden; 2 Department of Surgery, Skåne University Hospital, Malmö, Sweden; 3 Department of Clinical Epidemiology and Center for Population Medicine, Aarhus University, Aarhus, Denmark

**Keywords:** Colorectal cancer, Early-onset, Sweden, Follow-up

## Abstract

**Purpose:**

The incidence of colorectal cancer (CRC) is increasing in individuals aged < 50 years of age. This study aimed to examine whether high-frequency follow-up after CRC surgery reduces 5-year overall mortality, cancer-specific mortality and recurrence in patients with CRC aged ≤ 50 years.

**Methods:**

The COLOFOL trial performed between 2006 and 2010 was used to analyse patients randomised to high-frequency (computed tomography [CT] of the abdomen and thorax and a carcinoembryonic antigen [CEA] test at 6, 12, 18, 24 and 36 months) versus low-frequency (CT and CEA at 12 and 36 months) follow-up after curative CRC surgery. Intention-to-treat and per-protocol analyses were performed to study the primary outcomes (5-year overall mortality and cancer-specific mortality) and the secondary outcome (CRC recurrence), comparing the age groups ≤ 50, 51–70 and > 70 years.

**Results:**

In total, 2,509 patients were included in the intention-to-treat analysis with 183, 1,714 and 612 patients aged ≤ 50, 51–70 and > 70 years, respectively. The 5-year overall mortality risk for patients aged ≤ 50 was 8.3% in the high-frequency group compared with 8.4% in the low-frequency group (risk difference 0.2% [95% CI, − 8.0; 8.3]). The cancer-specific mortality risk for patients aged ≤ 50 years was 7.1% in the high-frequency group compared with 7.4% in the low-frequency group (risk difference, 0.3% [95% CI, − 7.4; 8.0]). The cancer-specific recurrence risk for patients aged ≤ 50 years was 12.9% in the high-frequency group compared with 21.0% in the low-frequency group (risk difference 8.1% [95% CI, − 2.6; 18.7]).

**Conclusion:**

Among individuals aged ≤ 50 years with stage II-III CRC, there was no reduction in overall mortality, cancer-specific mortality and cancer-specific recurrence with more intensive follow-up using CT and CEA.

**Supplementary Information:**

The online version contains supplementary material available at 10.1007/s00384-026-05096-9.

## Introduction

Colorectal cancer (CRC) is the third most prevalent malignancy in Europe, with nearly two-thirds of patients diagnosed with stage II or III disease [1–3]. Approximately 20% of patients experience recurrence, which is often associated with a poor prognosis [4]. In Sweden, the incidence of CRC is increasing in individuals aged < 50 years, regardless of tumour localisation [5, 6]. Studies indicate that patients aged < 50 years tend to be diagnosed at a more advanced tumour stage and often receive more intensive oncological treatment [7, 8]. These findings point towards a possible benefit of more intensive follow-up after surgery in this younger population. Additionally, a previous national cohort study in Sweden showed that patients with CRC aged < 50 years had a higher proportion of 30-day postoperative surgical complications, including anastomotic leakage, intraabdominal infections and wound infections compared with older age groups [9].

Over the past two decades, significant progress has been made in treating recurrent CRC, particularly through surgical resection of lung and liver metastasis along with enhanced adjuvant and palliative chemotherapy [10, 11]. Both historically and internationally, there have been variations in the frequency of patient follow-up after CRC surgery. However, the COLOFOL trial (NCT00225641) – conducted in Sweden, Denmark and Uruguay, involving CRC stage II or III patients under 76 years – found that increased follow-up testing frequency did not lead to a reduction in the 5-year mortality rate [12]. No studies have analysed the outcomes of different follow-up schedules based on age. The implementation of population-based CRC screening in Sweden has recently been fully rolled out and covers individuals aged 60–74 years. In Denmark, CRC screening was implemented gradually in 2014 for individuals aged 50–74 years [13]. Since 2018, the national public health authorities in Uruguay have recommended screening with immunochemical faecal occult blood test every two years for individuals aged 50–74 years [14]. This study aimed to examine if high-frequency follow-up after CRC surgery reduces 5-year overall mortality, cancer-specific mortality and cancer-specific recurrence in patients with CRC aged ≤ 50 years.

## Methods

All patients included in this study were selected from the COLOFOL trial cohort. The study design and methodology of the COLOFOL trial have been described in detail previously [12]. This study is a subgroup analysis and was not part of the initial study plan. The COLOFOL trial randomised 2,509 patients (1:1) with CRC across 24 hospitals in Denmark, Sweden and Uruguay to receive either low- or high-frequency follow-up between 2006 and 2010. The inclusion criteria comprised surgical resection with a curative intent (R0 resection) for colorectal adenocarcinoma; stage II (T3-4N0M0) or III (TanyN1-2M0); age under 76 years; a negative clean colon investigation within 3 months after surgery; and written consent from participants. Neoadjuvant treatment was allowed, according to national regimen protocols. The exclusion criteria included local resection for CRC; a clinical diagnosis of Lynch syndrome or familial adenomatous polyposis; a life expectancy less than 2 years due to concurrent diseases; refusal or inability to adhere to the follow-up regimens; participation in other clinical trials that could interfere with the follow-up regimens; and other or previous malignancies (excluding non-melanoma skin cancer). The study received ethical approval from committees in each participating country, including the Uppsala regional Ethical Review Board in Sweden (Dnr: 2004 M-453) and Frederiksberg and Copenhagen Scientific Committee in Denmark (KF 01–194/04). Written informed consent was obtained after the primary surgical CRC resection.

Patients randomised to the high-frequency follow-up group (experimental arm) underwent multi-slice contrast-enhanced computed tomography (CT) of the abdomen and thorax along with serum carcinoembryonic antigen (CEA) testing at 6, 12, 18, 24 and 36 months after surgery. In contrast, patients randomised to the low-frequency follow-up group (standard arm) underwent the same assessments only at 12 and 36 months after surgery. According to the study protocol, each centre was required to follow up on all participants with surveillance examinations for 3 years post-surgery. Moreover, they were expected to report any recurrence or death that occurred within 5 years after surgery. Following CRC resection, patients were monitored prospectively over a 5-year period. The primary outcomes assessed were the 5-year overall mortality rate and the cancer-specific mortality rate, while the secondary outcome was the 5-year cancer-specific recurrence rate.

## Statistical analysis

To examine the impact of follow-up frequency by age at CRC surgery, all patients were categorised into the age groups ≤ 50, 50–70 and > 70 years. Intention-to-treat and per-protocol analyses were performed to study 5-year overall mortality, cancer-specific mortality and recurrence across the age groups. There were missing recurrence data for 11 patients in the patients aged 50–70 and > 70 years (6 in the low-frequency follow-up group and 5 in the high-frequency follow-up group), which is why they were excluded from the recurrence and cancer-specific mortality analysis.

The incidence of primary and secondary outcomes was analysed using the cumulative incidence function and Gray’s test by age for between-group comparisons, accounting for competing risk where applicable. Risk differences were calculated from the cumulative incidence function estimates with 95% confidence intervals (CIs) in all age groups.

Crude and adjusted hazard ratios (HRs) with 95% CIs were computed using Cox proportional hazards regression analyses for the 5-year overall mortality, cancer-specific mortality and cancer-specific recurrence rates. The HRs were adjusted for sex, age at surgery, CRC location, stage, planned postoperative chemotherapy, history of diabetes, cardiovascular diseases, pulmonary diseases, cerebrovascular disease and smoking. The parallel hazard assumption was tested with log–log plots. The cumulative incidence curves suggested no significant difference between the low- and the high-frequency groups. According to that, log–log survival curves showed violation of the PH assumption for the overall analysis, thus HRs should be interpreted as average HR in the analysed follow-up period. The primary and secondary outcomes were further analysed using a frailty model with site as random factor to describe the influence of unobserved covariates due to the participation of various centres. The level of significance was set to P < 0.05. Statistical analyses were conducted using SAS version 9.4 (SAS Institute Inc). This study adheres to the STROBE (strengthening the reporting of observational studies in epidemiology) guidelines.

## Results

**Fig. 1:**
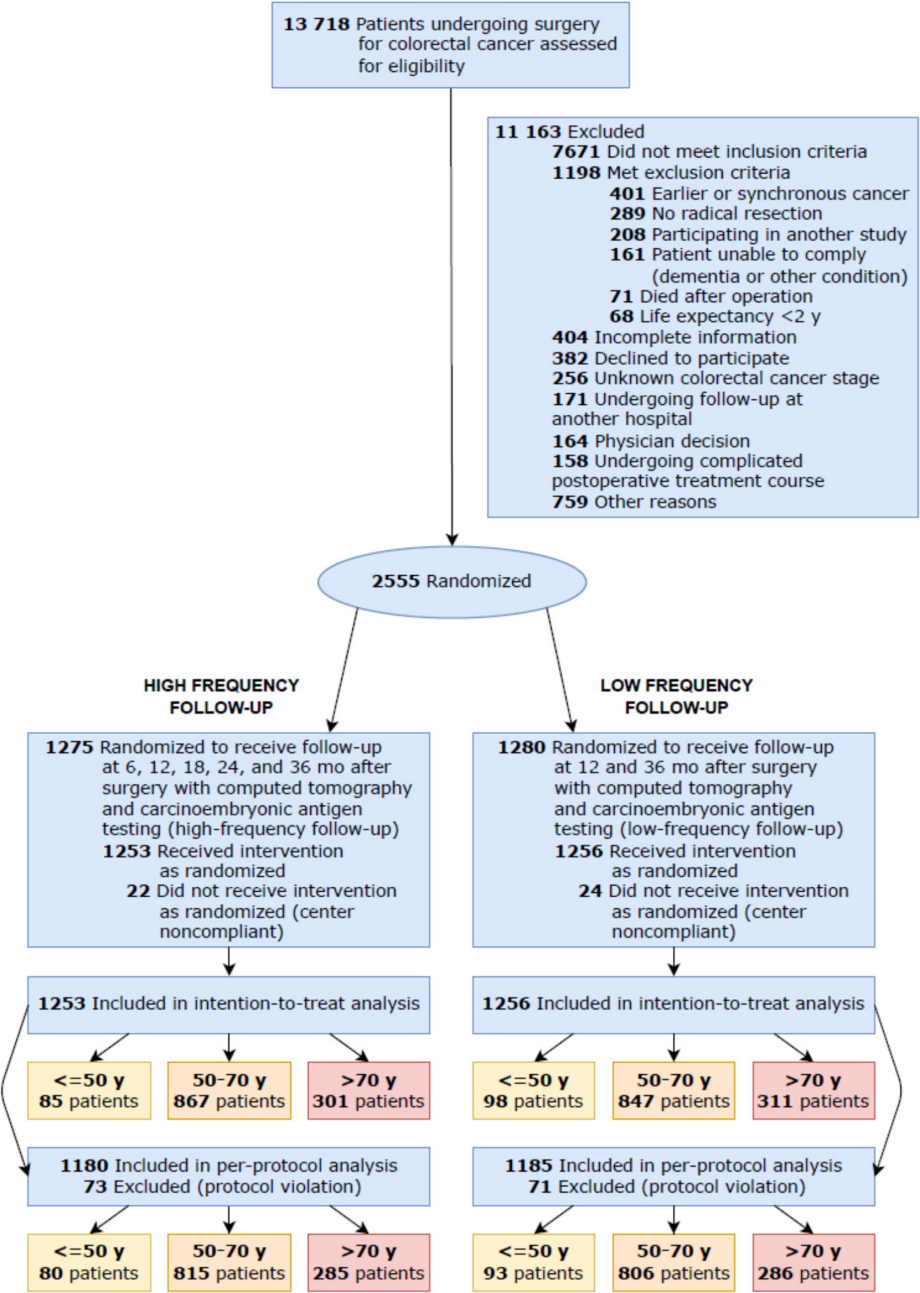
Flow chart diagram for inclusion of patients with CRC treated with curative resection between 2006–2010 and followed up

The study initially enrolled 13,718 patients who underwent surgery for CRC between 2006 and 2010. After exclusion, 2,555 patients were randomised, with a total of 2,509 included in the intention-to-treat analyses. These patients where further subdivided into three age groups: 183 patients were ≤ 50 years, 1,714 patients were 50–70 years and 612 patients were > 70 years. In the high-frequency follow-up, 73 patients (5.8%) were excluded due to protocol violations, while 71 patients (5.7%) were excluded in the low-frequency follow-up group (Fig. 1).

Table 1 presents the patient characteristics and clinical risk factors for the three age groups. Considering patients aged ≤ 50 years, the high- and low-frequency follow-up groups were well balanced across all variables except for cancer location, where colon cancer was more prevalent in the high-frequency follow-up group (68.2%) compared with the low-frequency follow-up group (50.0%). There was also a slight difference in daily smoking and alcohol consumption in patients aged ≤ 50 years, with a higher prevalence in the high-frequency follow-up group (20.0% and 25.9%, respectively) compared with the low-frequency follow-up group (14.3% and 16.3%). No differences were observed in the age groups 50–70 and > 70 years when comparing the two follow-up groups.

**Table 1:** Intention-to-treat analyses: Characteristics of patients with low-frequency follow-up and high frequency follow-up by age-group at date of operation

Age at the date of operation (years)									
	≤ 50, n (%)			51–70, n (%)			> 70, n (%)		
	Low F-U	High F-U	Total	Low F-U	High F-U	Total	Low F-U	High F-U	Total
Total	98 (100.0)	85 (100.0)	183 (100.0)	847 (100.0)	867 (100.0)	1714 (100.0)	311 (100.0)	301 (100.0)	612 (100.0)
Sex									
M	51 (52.0)	44 (51.8)	95 (51.9)	457 (54.0)	494 (57.0)	951 (55.5)	167 (53.7)	168 (55.8)	335 (54.7)
Cancer location									
Colon	49 (50.0)	58 (68.2)	107 (58.5)	539 (63.6)	565 (65.2)	1104 (64.4)	212 (68.2)	202 (67.1)	414 (67.6)
Rectum	49 (50.0)	27 (31.8)	76 (41.5)	308 (36.6)	302 (34.8)	610 (35.6)	99 (31.8)	99 (32.9)	198 (32.4)
TNM stage									
II (T3-4, N0, M0)	48 (49.0)	43 (50.6)	91 (49.7)	439 (51.8)	459 (52.9)	898 (52.4)	190 (61.1)	173 (57.5)	363 (59.3)
III (T1-4, N1-2, M0)	50 (51.0)	42 (49.4)	92 (50.3)	408 (48.2)	408 (47.1)	816 (47.6)	121 (38.9)	128 (42.5)	249 (40.7)
Dukes’ stage									
Dukes’ B	52 (53.1)	43 (50.6)	95 (51.9)	437 (51.6)	457 (52.7)	894 (52.2)	182 (58.5)	170 (56.5)	352 (57.5)
Dukes’ C	46 (46.9)	42 (49.4)	88 (48.1)	410 (48.4)	410 (47.3)	820 (47.8)	129 (41.5)	131 (43.5)	260 (42.5)
Diabetes									
Yes	2 (2.0)	2 (2.4)	4 (2.2)	70 (8.3)	87 (10.0)	157 (9.2)	35 (11.3)	28 (9.3)	63 (10.3)
Heart disease^†^									
Yes	8 (8.2)	7 (8.2)	15 (8.2)	269 (31.8)	246 (28.4)	515 (30.0)	137 (44.1)	130 (43.2)	267 (43.6)
Pulmonary disease									
Yes	0 (0)	3 (3.5)	3 (1.6)	41 (4.8)	52 (6.0)	93 (5.4)	25 (8.0)	22 (7.3)	47 (7.7)
Multiple sclerosis									
Yes	0 (0)	0 (0)	0 (0)	2 (0.2)	2 (0.2)	4 (0.2)	1 (0.3)	0 (0)	1 (0.2)
Cerebrovascular disease									
Yes	1 (1.0)	1 (1.2)	2 (1.1)	18 (2.1)	18 (2.1)	36 (2.1)	20 (6.4)	17 (5.6)	37 (6.0)
Other major disease									
Yes	3 (3.1)	1 (1.2)	4 (2.2)	39 (4.6)	42 (4.8)	81 (4.7)	16 (5.1)	16 (5.3)	32 (5.2)
Smoking									
Yes, daily	14 (14.3)	17 (20.0)	31 (16.9)	139 (16.4)	142 (16.4)	281 (16.4)	37 (11.9)	32 (10.6)	69 (11.3)
Yes, occasionally	2 (2.0)	0 (0)	2 (1.1)	18 (2.1)	5 (0.6)	23 (1.3)	2 (0.6)	2 (0.7)	4 (0.7)
No	73 (74.5)	63 (74.1)	136 (74.3)	650 (76.7)	674 (77.7)	1324 (77.2)	251 (80.7)	249 (82.7)	500 (81.7)
Unknown	9 (9.2)	5 (5.9)	14 (7.7)	40 (4.7)	46 (5.3)	86 (5.0)	21 (6.8)	18 (6.0)	39 (6.4)
Alcohol consumption									
Yes, < 3 drinks	15 (15.3)	19 (22.4)	34 (18.6)	184 (21.7)	175 (20.2)	359 (20.9)	67 (21.5)	58 (19.3)	125 (20.4)
Yes, ≥ 3 drinks	1 (1.0)	3 (3.5)	4 (2.2)	41 (4.8)	47 (5.4)	88 (5.1)	11 (3.5)	9 (3.0)	20 (3.3)
No	70 (71.4)	54 (63.5)	124 (67.8)	527 (62.2)	541 (62.4)	1068 (62.3)	194 (62.4)	202 (67.1)	396 (64.7)
Unknown	12 (12.2)	9 (10.6)	21 (11.5)	95 (11.2)	104 (12.0)	199 (11.6)	39 (12.5)	32 (10.6)	71 (11.6)

F-U follow-up

† Includes acute myocardial infarction, hypertension and other heart disease

**Fig. 2:**
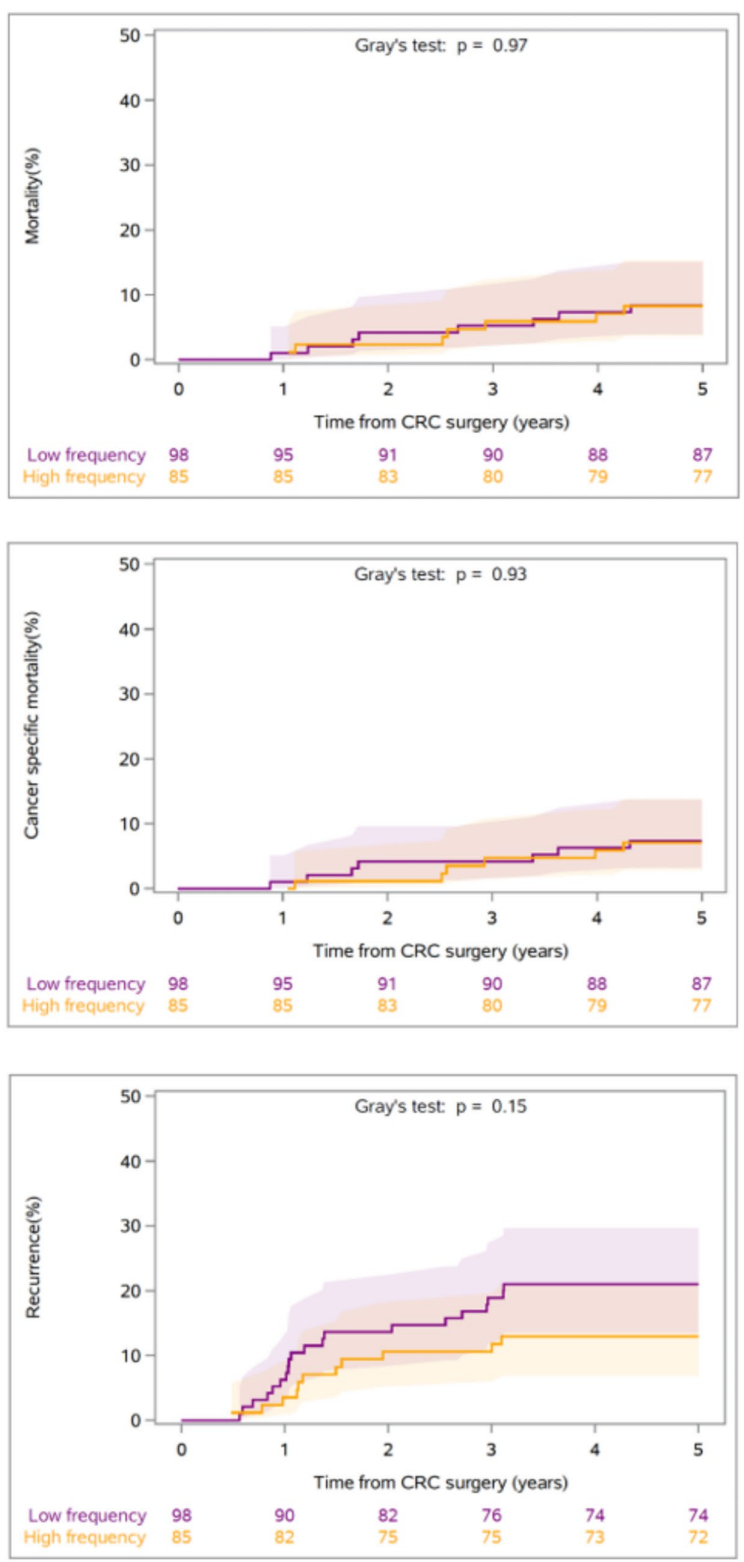
5-year overall mortality, cancer-specific mortality and cancer-specific recurrence by time from CRC surgery for patients ≤ 50 years in the intention-to-treat analysis

Among the 183 patients aged ≤ 50 years included in the intention-to-treat analysis, the 5-year overall mortality risk was 8.3% (95% CI 3.6%; 15.4%) in the high-frequency follow-up group compared with 8.4% (95% CI 3.9%; 15.1%) in the low-frequency follow-up group (risk difference 0.2% [95% CI − 8.0%; 8.3%]) (Table 2). The cumulative incidence function for the 5-year overall mortality risk in patients ≤ 50 years is plotted in Fig. 2, where the shaded areas represent the 95% CIs.

**Table 2:** Intention-to-treat: 5-year overall mortality risk, cancer-specific mortality risk, cancer-specific recurrence risk

Outcome	Age group	Risk in the low frequency group (95% CI)	Risk in the high frequency group (95% CI)	Risk difference
Overall mortality	All	14.1 (12.2;16.1)	13.0 (11.2;15.0)	1.1 (−1.6;3.8)
Overall mortality	≤ 50 years	8.4 (3.9;15.1)	8.3 (3.6;15.4)	0.2 (−8.0;8.3)
Overall mortality	51—70 years	13.0 (10.9;15.4)	12.4 (10.3;14.7)	0.6 (−2.6;3.8)
Overall mortality	> 70 years	18.9 (14.7;23.6)	16.3 (12.3;20.7)	2.7 (−3.4;8.8)
Cancer-specific mortality	All	11.2 (9.5;13.0)	10.4 (8.8;12.2)	0.8 (−1.7;3.2)
Cancer-specific mortality	≤ 50 years	7.4 (3.2;13.8)	7.1 (2.9;13.9)	0.3 (−7.4;8.0)
Cancer-specific mortality	51—70 years	10.8 (8.8;13.0)	10.1 (8.2;12.3)	0.7 (−2.3;3.6)
Cancer-specific mortality	> 70 years	13.4 (9.8;17.6)	12.2 (8.8;16.2)	1.2 (−4.2;6.6)
Recurrence	All	19.3 (17.1;21.5)	21.4 (19.2;23.7)	−2.2 (−5.3;1.0)
Recurrence	≤ 50 years	21.0 (13.4;29.7)	12.9 (6.8;21.0)	8.1 (−2.6;18.7)
Recurrence	51—70 years	18.6 (16.1;21.3)	22.3 (19.5;25.1)	−3.6 (−7.5;0.2)
Recurrence	> 70 years	20.5 (16.2;25.2)	21.5 (17.0;26.3)	−1.0 (−7.5;5.6)

In the intention-to-treat analysis, the cancer-specific mortality risk for patients aged ≤ 50 years was 7.1% (95% CI 2.9%; 13.9%) in the high-frequency follow-up group versus 7.4% (95% CI 3.2%; 13.8%) in the low-frequency follow-up group (risk difference 0.3% [95% CI − 7.4%; 8.0%]) (Table 2). The cumulative incidence function for the cancer-specific mortality risk in patients ≤ 50 years is presented in Fig. 2.

The cancer-specific recurrence risk for patients aged ≤ 50 years in the intention-to-treat analysis was 13.0% (95% CI 6.8%; 21.0%) in the high-frequency follow-up group compared with 21.0% (95% CI 13.4%; 29.7%) in the low-frequency follow-up group (risk difference 8.1% [95% CI − 2.6%; 18.7%]) (Table 2). When analysing the patients aged 50–70 and > 70 years, the cancer-specific recurrence risk, although not significant, was higher for the high-frequency follow-up group during the periods when the low-frequency follow-up group had no follow-ups (6 to 12 months and 18 to 36 months) (Figure S5 and S6). However, this trend was not observed in the ≤ 50 years age group where the high-frequency follow-up consistently had a lower cancer-specific recurrence risk throughout the entire follow-up period (Fig. 2).

There was no significant difference between the two follow-up groups when analysing the 5-year overall mortality risk, cancer-specific mortality risk and 5-year cancer-specific recurrence risk in the patients aged 50–70 and > 70 years in the intention-to-treat analyses (Table 2).

The intention-to-treat analysis indicated no associations in the Cox regression analysis, after adjusting for confounders, between the high- and low-frequency follow-up groups regarding the 5-year overall mortality rate (adjusted HR 1.2 [95% CI 0.4; 3.5]) or the cancer-specific mortality rate (adjusted HR 1.3 [95% CI 0.4; 4.4]) for patients aged ≤ 50 years. Additionally, the risk of cancer-specific recurrence did not significantly differ between the high- and low-frequency follow-up groups (adjusted HR 0.7 [95% CI 0.3; 1.5]). The frailty analysis also revealed no differences between the groups (Table 3).

**Table 3:** Intention-to-treat: Cox regression analyses

Outcome	Age group	Crude HR when comparing high-vs. low-frequency groups	Adjusted HR when comparing high- vs low-frequency groups	Frailty model: crude HR with site as a RANDOM EFFECT	Frailty model: adjusted HR with site as a RANDOM EFFECT
Overall mortality	All	0.9 (0.7;1.1)	0.9 (0.7;1.1)	0.9 (0.7;1.1)	0.9 (0.7;1.1)
Overall mortality	≤ 50 years	1.0 (0.4;2.7)	1.2 (0.4;3.5)	1.0 (0.4;2.7)	1.2 (0.4;3.7)
Overall mortality	51—70 years	0.9 (0.7;1.2)	0.9 (0.7;1.2)	0.9 (0.7;1.2)	0.9 (0.7;1.2)
Overall mortality	> 70 years	0.9 (0.6;1.3)	0.8 (0.6;1.2)	0.9 (0.6;1.3)	0.8 (0.6;1.2)
Cancer-specific mortality	All	0.9 (0.7;1.2)	0.9 (0.7;1.2)	0.9 (0.7;1.2)	0.9 (0.7;1.2)
Cancer-specific mortality	≤ 50 years	1.0 (0.3;2.9)	1.3 (0.4;4.4)	1.0 (0.3;2.9)	1.3 (0.4;4.3)
Cancer-specific mortality	51—70 years	0.9 (0.7;1.3)	0.9 (0.7;1.2)	0.9 (0.7;1.3)	0.9 (0.7;1.2)
Cancer-specific mortality	> 70 years	0.9 (0.6;1.4)	0.9 (0.6;1.4)	0.9 (0.6;1.4)	0.9 (0.6;1.4)
Recurrence	All	1.1 (1.0;1.4)	1.2 (1.0;1.4)	1.1 (1.0;1.4)	1.2 (1.0;1.4)
Recurrence	≤ 50 years	0.6 (0.3;1.2)	0.7 (0.3;1.5)	0.6 (0.3;1.2)	0.7 (0.3;1.5)
Recurrence	51—70 years	1.2 (1.0;1.5)	1.3 (1.0;1.6)	1.2 (1.0;1.5)	1.3 (1.0;1.6)
Recurrence	> 70 years	1.1 (0.8;1.5)	1.1 (0.7;1.5)	1.1 (0.8;1.5)	1.1 (0.7;1.5)

The results of the per-protocol analyses are presented in the supplementary files.

## Discussion

Significant advancements have been made in managing CRC recurrences, but previous studies have not demonstrated that intensified follow-up testing has a survival benefit. However, there are no large studies examining the subgroup of younger patients, where a benefit might be more detectable. This study analysed data from 2,509 patients treated for CRC who participated in a multicentre randomised trial. We evaluated the impact of low-frequency versus high-frequency follow-up testing, focusing on age-related differences. We found no differences in the 5-year overall mortality, cancer-specific mortality or cancer-specific recurrence rates when comparing the follow-up groups after CRC surgery in patients aged ≤ 50 years.

When the COLOFOL trial began in 2005, Sweden had no standardised surveillance programmes, and follow-up varied widely from annual check-ups to no follow-up during 5 years. In 2014, the national guidelines by the Swedish National Board of Health and Welfare did not provide specific recommendations, only noting that studies were ongoing. However, in 2016 the Swedish national guidelines for CRC recommended the low-frequency regimen as an interim measure [15]. In 2005, Danish guidelines recommended follow-up similar to the low-frequency follow-up group in the COLOFOL trial [16], and no national guidelines were in place in Uruguay. Although some studies have indicated that more frequent follow-up can lead to earlier detection of recurrences [17, 18], none has shown a survival benefit from such intensive follow-up. The current surveillance programmes in Sweden and Denmark, guided by findings from the COLOFOL, FACS and NCDB studies [12, 17, 19], recommend low-frequence follow-up at 12 and 36 months after CRC surgery.

There is still some uncertainty regarding whether specific subgroups, such as high-risk patients who may benefit from aggressive treatment of recurrences, would benefit from more frequent monitoring involving CT and CEA at 6, 12, 18, 24 and 36 months post-surgery. Patient selection for such follow-up protocols is typically determined during postoperative multidisciplinary meetings. In a recent Danish nationwide cohort study encompassing 25,729 patients, Nors et al. demonstrated that early-onset CRC was associated with a higher 5-year cumulative incidence of recurrence (29%) compared with late-onset CRC (21%) across all disease stages (I-III), especially during the first year after surgery [20]. Given that younger patients often have fewer comorbidities and can tolerate more aggressive treatments for recurrences than older patients, a compelling question is whether follow-up schedules should be adapted to reflect this. An outcome study after colorectal liver metastases within the COLOFOL trial revealed that a majority of patients (76%) were treated with a curative intent achieving a 5-year overall survival rate of 60% [21]. The authors also found that patients treated for liver metastases had a significantly better 8-year overall survival if they were randomised to the high-frequency follow-up group compared with those in the low-intensity follow-up group. These findings underscore the need for additional studies to investigate whether intensified follow-up is warranted for high-risk patients available for further treatment [21]. In younger patients, often assumed to benefit more from early detection due to their longer life expectancy and generally better performance status, our findings suggest that the testing frequency may not drive better long-term outcomes. Instead, a shift towards personalised follow-up strategies incorporating molecular profiling and tumour biology may provide more meaningful clinical benefits than age-based surveillance protocols alone. With early-onset CRC on the rise, future research should explore risk-tailored surveillance over simply increasing surveillance intensity.

The primary strength of this study is the prospectively collected data of 2, 509 patients within a randomised clinical trial, with an inclusion rate of 56.4%, supporting the generalisability of the results [12, 22]. However, there are several limitations. One is the lack of age-stratified randomisation, making this a subgroup analysis that complicates interpretation of the findings and leaves the study underpowered. Consequently, the results should be interpreted carefully. Moreover, the subgroup of patients aged ≤ 50 years comprised only 183 individuals in the intention-to-treat analysis, which limits the statistical power to detect potentially meaningful differences in outcomes, leading to potential imprecision due to the small sample size. While our findings did not demonstrate a statistically significant difference in outcomes between the follow-up intensities in patients aged ≤ 50 years, this study was not sufficiently powered to definitely rule out potential differences. Therefore, these results should be interpreted as hypothesis generating and warrant further validation in larger, adequately powered cohorts. The exclusion of patients older than 76 years in the COLOFOL trial limits the representation of patients aged > 70 years who might be suitable candidates for follow-up and aggressive treatment of recurrences when comorbidity is low. The higher recurrence rate in patients aged ≤ 50 years in the low-intensity follow-up group, despite no survival difference, should be interpreted cautiously. Given the limited sample size in this subgroup, this finding may reflect confounding or bias. Additionally, although recurrence rates can differ between colon and rectal cancers, our sample size did not permit meaningful stratification by tumour location which is why we could not explore potential differences in follow-up efficacy between these subgroups.

This study showed no reduction in overall mortality, cancer-specific mortality and cancer-specific recurrence with intensive follow-up using CT and CEA in patients aged ≤ 50 years with stage II-III CRC. Current surveillance strategies for early-onset CRC have been insufficiently studied, highlighting the need for future studies to determine whether these patients could benefit from more intensive or personalised monitoring.

## Supplementary Information

Below is the link to the electronic supplementary material.

ESM 1 DOCX (1.24 MB)

## Data Availability

The data supporting the findings of this study are not publicly available due to ethical constrains and can therefore not be shared openly.

## References

[1] Ferlay J, Steliarova-Foucher E, Lortet-Tieulent J, Rosso S, Coebergh JW, Comber H, et al. Cancer incidence and mortality patterns in Europe: estimates for 40 countries in 2012. European journal of cancer (Oxford, England : 1990). 2013;49(6):1374–403. 10.1016/j.ejca.2012.12.027 23485231

[2] Swedish Colorectal Cancer Registry, Quality Report, Colon Cancer. https://cancercentrumse/globalassets/cancerdiagnoser/tjock--och-andtarm-anal/kvalitetsregister/tjock--och-andtarm-2022/kolonrapport2021_2022-05-23pdf . 2021.

[3] Swedish Colorectal Cancer Registry, Quality Report, Rectal Cancer https://cancercentrumse/globalassets/cancerdiagnoser/tjock--och-andtarm-anal/kvalitetsregister/tjock--och-andtarm-2022/rektalrapport2021_2022-05-24pdf 2021.

[4] Nors J, Iversen LH, Erichsen R, Gotschalck KA, Andersen CL (2024) Incidence of recurrence and time to recurrence in stage I to III colorectal cancer: a nationwide Danish cohort study. JAMA Oncol 10(1):54–62 37971197 10.1001/jamaoncol.2023.5098 PMC10654928

[5] Barot S, Liljegren A, Nordenvall C, Blom J, Radkiewicz C (2025) Incidence trends and long-term survival in early-onset colorectal cancer: a nationwide Swedish study. Annals of oncology : official journal of the European Society for Medical Oncology 36(11):1400–1408 40816336 10.1016/j.annonc.2025.07.019

[6] Gutlic I, Schyman T, Lydrup M-L, Buchwald P (2019) Increasing colorectal cancer incidence in individuals aged < 50 years—a population-based study. Int J Colorectal Dis 34(7):1221–1226 31102007 10.1007/s00384-019-03312-3

[7] Saraste D, Järås J, Martling A (2020) Population-based analysis of outcomes with early-age colorectal cancer. Br J Surg 107(3):301–309 31925793 10.1002/bjs.11333

[8] Zaborowski AM, Abdile A, Adamina M, Aigner F, d’Allens L, Allmer C et al (2021) Characteristics of Early-Onset vs Late-Onset Colorectal Cancer: A Review. JAMA Surg 156(9):865–874 34190968 10.1001/jamasurg.2021.2380

[9] Gutlic I, Saraste D, Nordenvall C, Martling A, Lydrup ML, Buchwald P (2024) Postoperative complications and emergency surgeries in colorectal cancer patients <50 years-a national cohort study. Colorectal disease : the official journal of the Association of Coloproctology of Great Britain and Ireland 26(7):1397–1404 38858822 10.1111/codi.17058

[10] Kanas GP, Taylor A, Primrose JN, Langeberg WJ, Kelsh MA, Mowat FS et al (2012) Survival after liver resection in metastatic colorectal cancer: review and meta-analysis of prognostic factors. Clin Epidemiol. 10.2147/CLEP.S34285 23152705 10.2147/CLEP.S34285 PMC3496330

[11] Hansdotter P, Scherman P, Petersen SH, Mikalonis M, Holmberg E, Rizell M et al (2021) Patterns and resectability of colorectal cancer recurrences: outcome study within the COLOFOL trial. BJS Open. 10.1093/bjsopen/zrab067 34308474 10.1093/bjsopen/zrab067 PMC8311321

[12] Wille-Jørgensen P, Syk I, Smedh K, Laurberg S, Nielsen DT, Petersen SH et al (2018) Effect of More vs Less Frequent Follow-up Testing on Overall and Colorectal Cancer-Specific Mortality in Patients With Stage II or III Colorectal Cancer: The COLOFOL Randomized Clinical Trial. JAMA 319(20):2095–2103 29800179 10.1001/jama.2018.5623 PMC6583244

[13] Danish Colorectal Cancer Group. Danish Colorectal Cancer Group website. https://dccgdk/wp-content/uploads/2023/07/2014_screeningpdf . Accessed October 29, 2024.

[14] Reich M, Buki LP (2021) Colorectal cancer screening in Uruguay: current assessment and roadmap for the future. Psicol Reflex Crit 34(1):20 34185179 10.1186/s41155-021-00178-9 PMC8241939

[15] Swedish Colorectal Cancer Registry. National guidlines in Sweden. https://kunskapsbankencancercentrumse/diagnoser/tjock-och-andtarmscancer/vardprogram/ . Accessed October 29, 2024.

[16] Danish Colorectal Cancer group. Danish Colorectal Cancer Group Website. . https://dccgdk/arsrapporter/ . Accessed October 29, 2024.

[17] Primrose JN, Perera R, Gray A, Rose P, Fuller A, Corkhill A et al (2014) Effect of 3 to 5 years of scheduled CEA and CT follow-up to detect recurrence of colorectal cancer: the FACS randomized clinical trial. JAMA 311(3):263–270 24430319 10.1001/jama.2013.285718

[18] Verberne CJ, Zhan Z, van den Heuvel E, Grossmann I, Doornbos PM, Havenga K et al (2015) Intensified follow-up in colorectal cancer patients using frequent Carcino-Embryonic Antigen (CEA) measurements and CEA-triggered imaging: results of the randomized “CEAwatch” trial. European Journal of Surgical Oncology (EJSO) 41(9):1188–1196 26184850 10.1016/j.ejso.2015.06.008

[19] Snyder RA, Hu CY, Cuddy A, Francescatti AB, Schumacher JR, Van Loon K et al (2018) Association between intensity of posttreatment surveillance testing and detection of recurrence in patients with colorectal cancer. JAMA 319(20):2104–2115 29800181 10.1001/jama.2018.5816 PMC6151863

[20] Nors J, Gotschalck KA, Erichsen R, Andersen CL (2024) Risk of recurrence in early-onset versus late-onset non-metastatic colorectal cancer, 2004–2019: a nationwide cohort study. Lancet Reg Health Am. 10.1016/j.lanepe.2024.101093 10.1016/j.lanepe.2024.101093 PMC11483332 39421193

[21] Scherman P, Hansdotter P, Holmberg E, Viborg Mortensen F, Petersen SH, Rizell M et al (2023) High resection rates of colorectal liver metastases after standardized follow-up and multimodal management: an outcome study within the COLOFOL trial. HPB 25(7):766–774 36967324 10.1016/j.hpb.2023.03.003

[22] Hansdotter Andersson P, Wille-Jørgensen P, Horváth-Puhó E, Petersen SH, Martling A, Sørensen HT et al (2016) The COLOFOL trial: study design and comparison of the study population with the source cancer population. Clin Epidemiol 8:15–21 26869813 10.2147/CLEP.S92661 PMC4734721

