# Supplementary material for: Follow-up intensity after colorectal cancer surgery in patients aged ≤ 50, 50–70 and > 70 years – an analysis within the COLOFOL randomised clinical trial
Source: Int J Colorectal Dis. 2026 Jan 26;41(1):47. doi: 10.1007/s00384-026-05096-9 (PMC12835091; doi:10.1007/s00384-026-05096-9)

**Follow-up intensity after colorectal cancer surgery in patients aged ≤50 years – an analysis within the COLOFOL randomized clinical trial**

Ida Gutlic^1,2^, Katalin Veres^3^, Erzsébet Horváth-Puhó^3^, Marie-Louise Lydrup^1,2^, Pamela Buchwald^1,2^ on behalf of the COLOFOL-study group.

^1^Department of Clinical Sciences Malmö, Lund University, Malmö, Sweden

^2^Department of Surgery, Skåne University Hospital

^3^Department of Clinical Epidemiology and Center for Population Medicine, Aarhus University, Aarhus, Denmark

**Corresponding author;** Ida Gutlic

**ORCID ID**; 0000-0003-0093-9766

**Supplementary Materials - Index**

| **Supplementary Figures and Tables** |  |
| --- | --- |
| Intention-to-treat figures – 5-year overall mortality, cancer-specific mortality, cancer-specific recurrence rate (all age-groups) | *pag. 2* |
| Intention-to-treat figures – 5-year overall mortality rate (51-70 & >70 years) | *pag. 3* |
| Intention-to-treat figures – cancer-specific mortality rate (51-70 & >70 years) | *pag. 4* |
| Intention-to-treat figures – cancer-specific recurrence rate (51-70 & >70 years) | *pag. 5* |
| Per-protocol table– patient characteristics | *pag. 6* |
| Per-protocol table – 5-year overall mortality, cancer-specific mortality, cancer-specific recurrence risk | *pag. 7* |
| Per-protocol table – Cox regression analysis | *pag. 8* |
| Per-protocol figures – 5-year overall mortality, cancer-specific mortality, cancer-specific recurrence rate (all age-groups) | *pag. 9* |
| Per-protocol figures – 5-year overall mortality rate (≤50, 51-70 & >70 years) | *pag. 10* |
| Per-protocol figures – cancer-specific mortality rate (≤50, 51-70 & >70 years) | *pag. 11* |
| Per-protocol figures – cancer-specific recurrence rate (≤50, 51-70 & >70 years) | *pag. 12* |
|  |  |

**Supplementary Figures and Tables**

**Fig. 1** Intention-to-treat: 5-year overall mortality rate for all age-groups


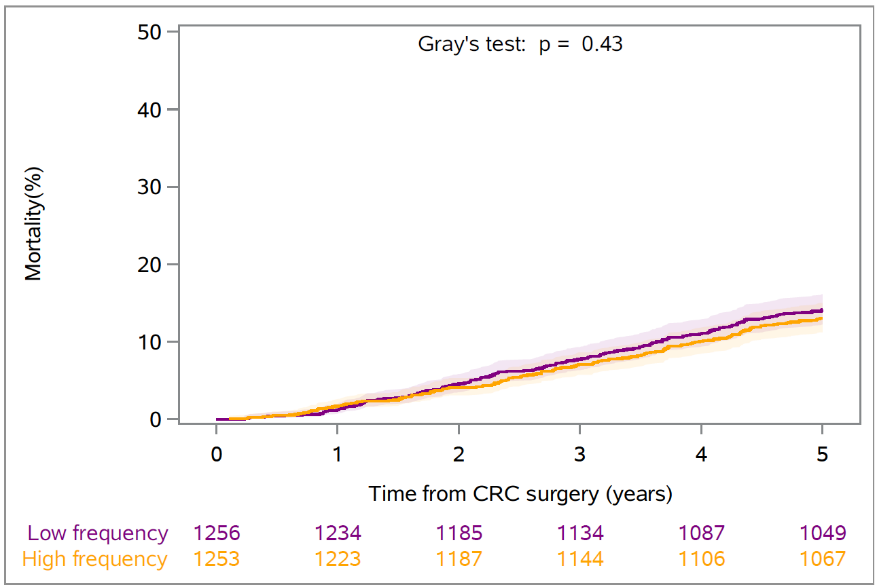


**Fig. 2** Intention-to-treat: cancer-specific mortality rate for all age-groups


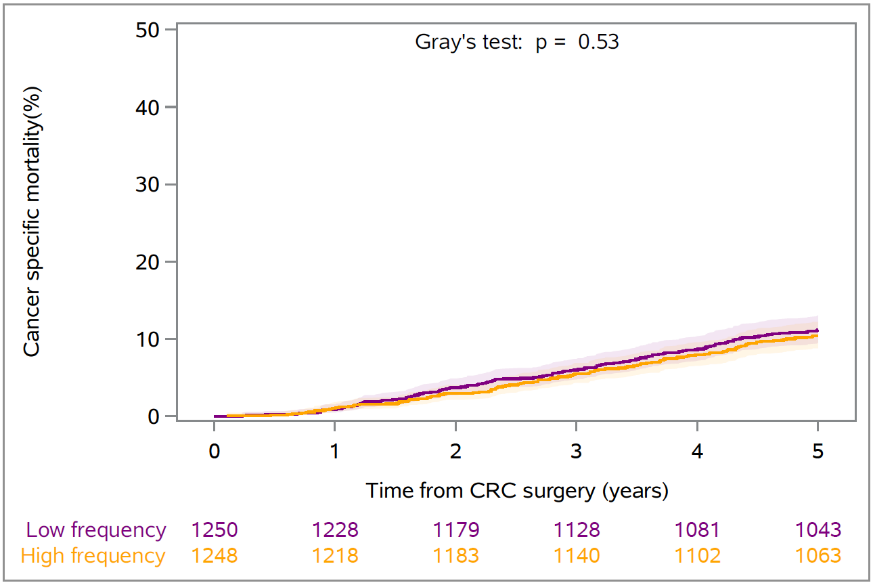


**Fig. 3** Intention-to-treat: cancer-specific recurrence rate for all age-groups


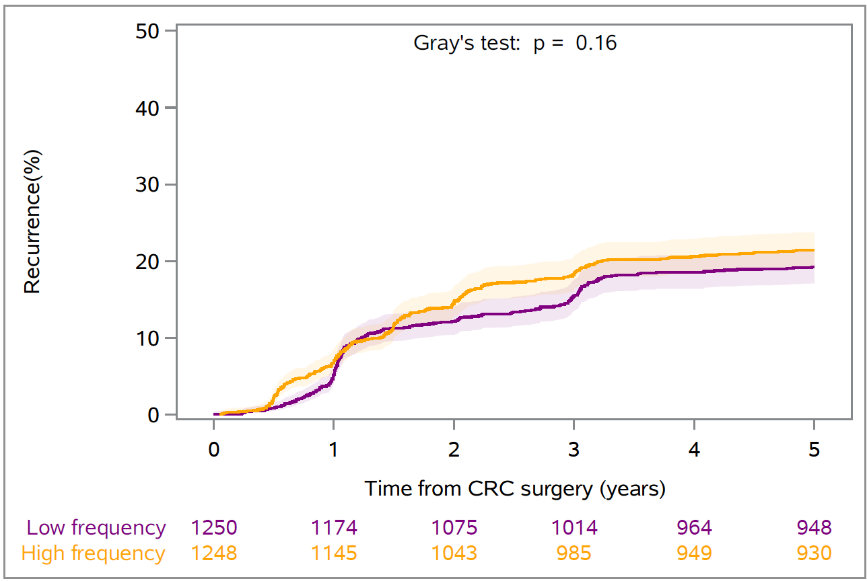


**Fig. 4** Intention-to-treat: 5-year overall mortality rate for patients 51-70 years


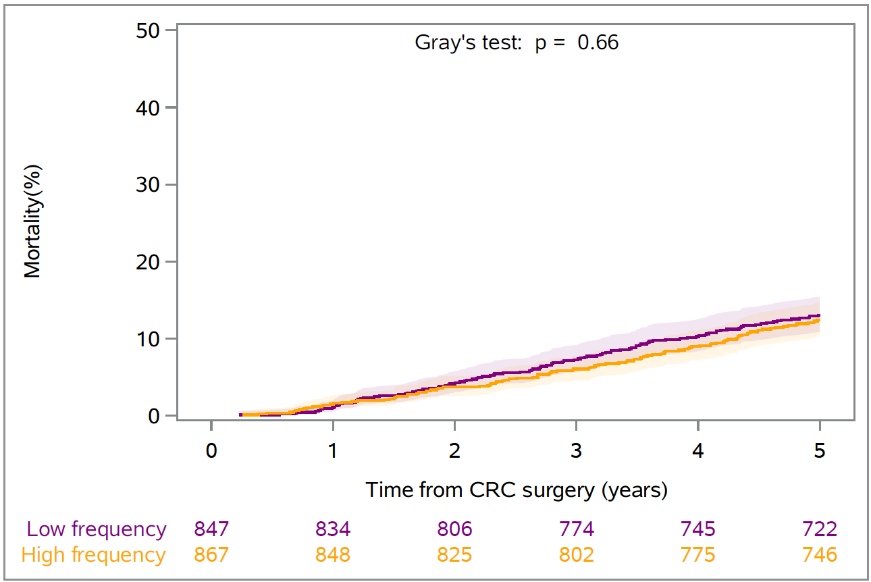


**Fig. 5** Intention-to-treat: 5-year overall mortality rate for patients > 70 years


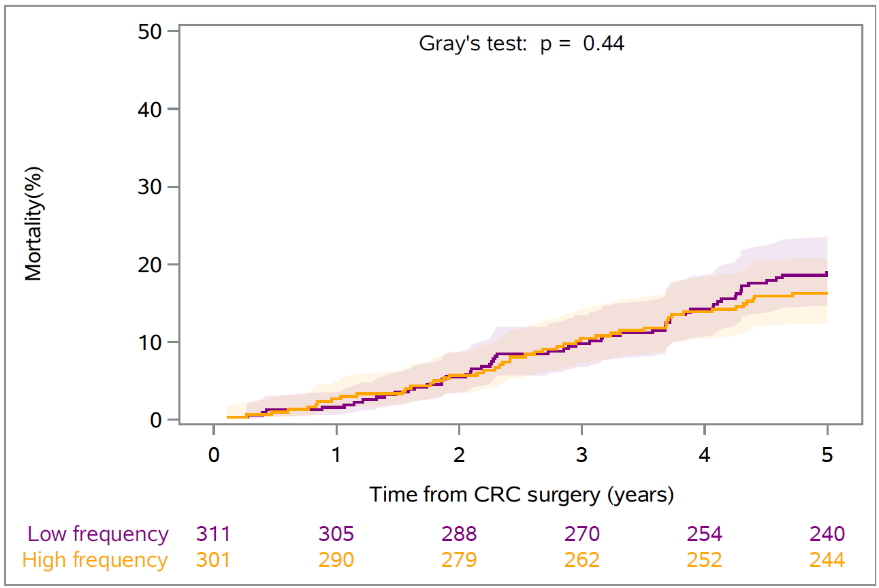


**Fig. 6** Intention-to-treat: cancer-specific mortality rate for patients 51-70 years


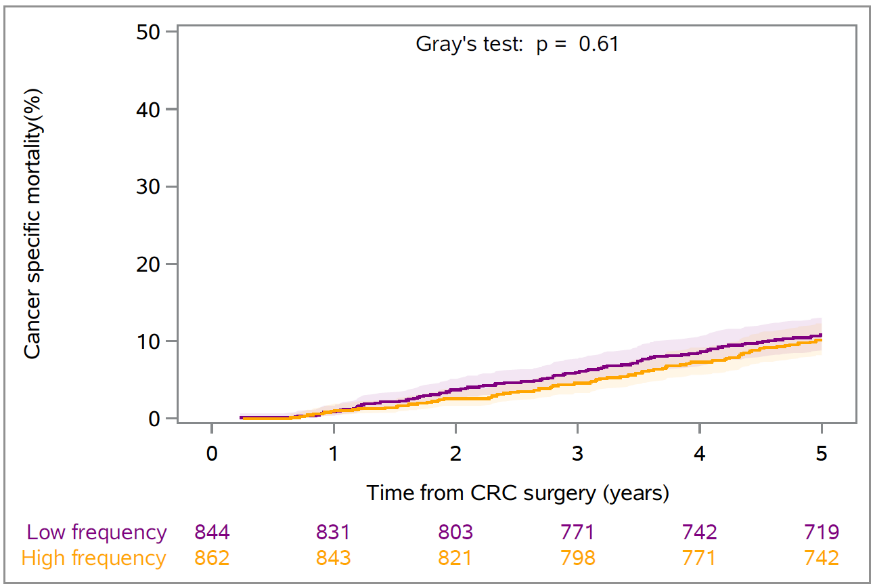


**Fig. 7**. Intention-to-treat: cancer-specific mortality rate for patients > 70 years


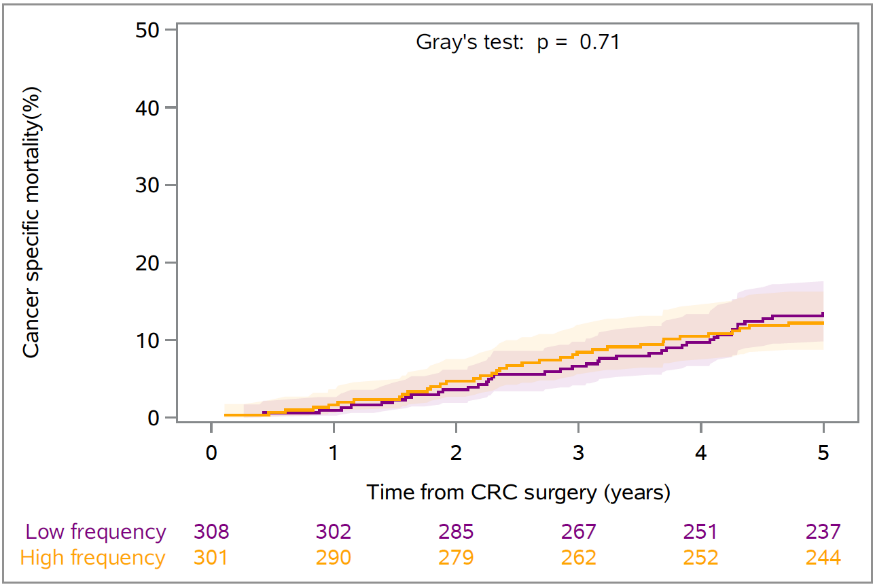


**Fig. 8** Intention-to-treat: cancer-specific recurrence rate for patients 51-70 years


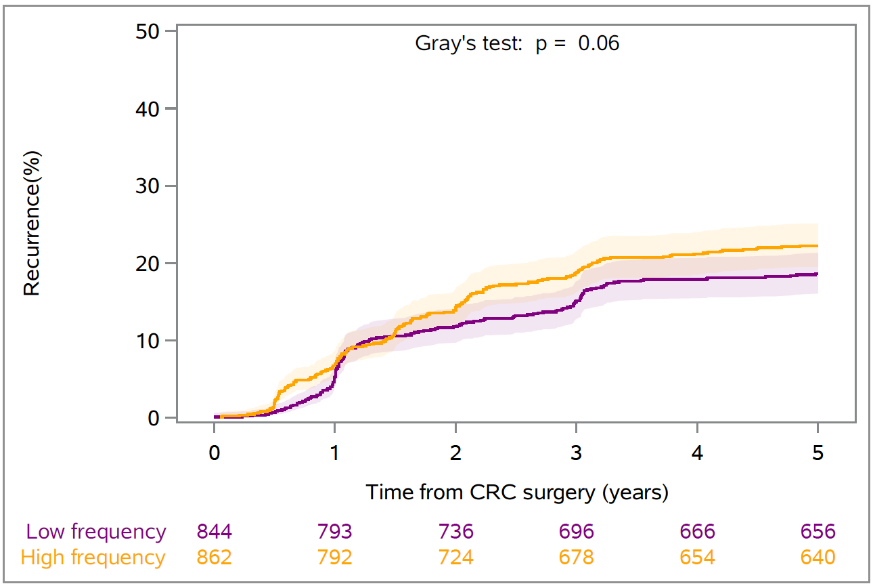


**Fig. 9** Intention-to-treat: cancer-specific recurrence rate for patients > 70 years


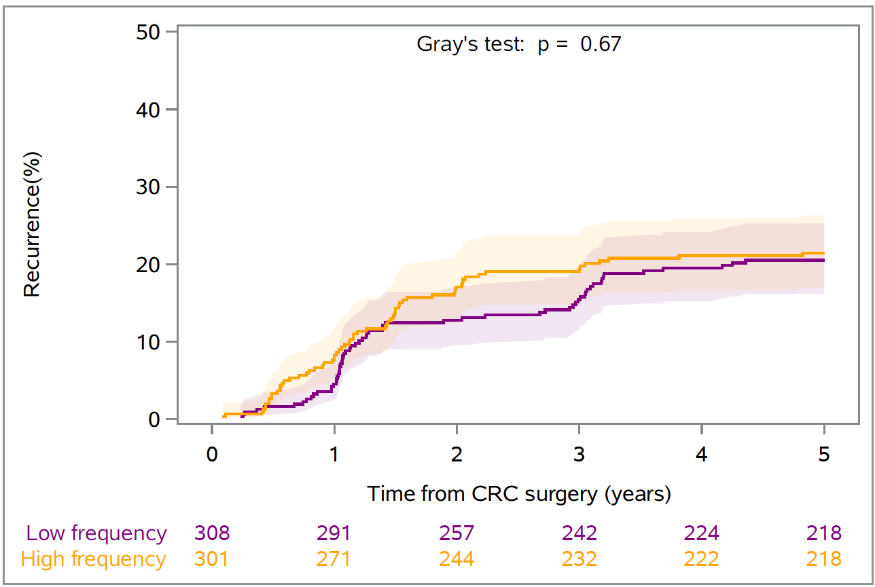


**Table 1** Per-protocol analyses: Characteristics of patients with low-frequency follow-up and high frequency follow-up by age-group at date of operation

| **Age at date of operation (years)**  ≤ **50, n (%)** **51-70, n (%)** **> 70, n (%)**  Low F-U High F-U Total Low F-U High F-U Total Low F-U High F-U Total |
| --- |
| **Total** 93 (100.0) 80 (100.0) 173 (100.0) 806 (100.0) 815 (100.0) 1621 (100.0) 286 (100.0) 285 (100.0) 571 (100.0) |
| **Sex** |
| M 49 (52.7) 40 (50.0) 89 (51.4) 432 (53.6) 464 (56.9) 896 (55.3) 153 (53.5) 159 (55.8) 312 (54.6) |
| **Cancer location** |
| Colon 45 (48.4) 55 (68.8) 100 (57.8) 513 (63.6) 525 (64.4) 1038 (64.0) 194 (67.8) 188 (66.0) 382 (66.9) |
| Rectum 48 (51.6) 25 (31.3) 73 (42.2) 293 (36.4) 290 (35.6) 583 (36.0) 92 (32.2) 97 (34.0) 189 (33.1) |
| **TNM stage** |
| II (T3-4, N0, M0) 44 (47.3) 43 (53.8) 87 (50.3) 420 (52.1) 420 (51.5) 840 (51.8) 177 (61.9) 162 (56.8) 339 (59.4) |
| III (T1-4, N1-2, M0) 49 (52.7) 37 (46.3) 86 (49.7) 386 (47.9) 395 (48.5) 781 (48.2) 109 (38.1) 123 (43.2) 232 (40.6) |
| **Dukes’ stage** |
| Dukes’ B 48 (51.6) 43 (53.8) 91 (52.6) 418 (51.9) 420 (51.5) 838 (51.7) 169 (59.1) 159 (55.8) 328 (57.4) |
| Dukes’ C 45 (48.4) 37 (46.3) 82 (47.4) 388 (48.1) 395 (48.5) 783 (48.3) 117 (40.9) 126 (44.2) 243 (42.6) |
| **Diabetes** |
| Yes 2 (2.2) 2 (2.5) 4 (2.3) 65 (8.1) 81 (9.9) 146 (9.0) 32 (11.2) 25 (8.8) 57 (10.0) |
| **Heart dis.**^†^ |
| Yes 8 (8.6) 7 (8.8) 15 (8.7) 250 (31.0) 231 (28.3) 481 (29.7) 126 (44.1) 125 (43.9) 251 (44.0) |
| **Pulmonary dis.** |
| Yes 0 (0) 2 (2.5) 2 (1.2) 36 (4.5) 47 (5.8) 83 (5.1) 24 (8.4) 20 (7.0) 44 (7.7) |
| **Multiple sclerosis** |
| Yes 0 (0) 0 (0) 0 (0) 1 (0.1) 1 (0.1) 2 (0.1) 1 (0.3) 0 (0) 1 (0.2) |
| **Cerebrovascular dis.** |
| Yes 1 (1.1) 1 (1.3) 2 (1.2) 17 (2.1) 17 (2.1) 34 (2.1) 18 (6.3) 16 (5.6) 34 (6.0) |
| **Other major dis.** |
| Yes 3 (3.2) 1 (1.3) 4 (2.3) 38 (4.7) 41 (5.0) 79 (4.9) 15 (5.2) 16 (5.6) 31 (5.4) |
| **Smoking** |
| Yes, daily 12 (12.9) 14 (17.5) 26 (15.0) 136 (16.9) 133 (16.3) 269 (16.6) 34 (11.9) 31 (10.9) 65 (11.4) |
| Yes, occasionally 2 (2.2) 0 (0) 2 (1.2) 16 (2.0) 5 (0.6) 21 (1.3) 1 (0.3) 2 (0.7) 3 (0.5) |
| No 70 (75.3) 61 (76.3) 131 (75.7) 616 (76.4) 635 (77.9) 1251 (77.2) 235 (82.2) 234 (82.1) 469 (82.1) |
| Unknown 9 (9.7) 5 (6.3) 14 (8.1) 38 (4.7) 42 (5.2) 80 (4.9) 16 (5.6) 18 (6.3) 34 (6.0) |
| **Alcohol consumption** |
| Yes, < 3 drinks 15 (16.1) 17 (21.3) 32 (18.5) 178 (22.1) 166 (20.4) 344 (21.2) 57 (19.9) 56 (19.6) 113 (19.8) |
| Yes, ≥ 3 drinks 1 (1.1) 3 (3.8) 4 (2.3) 40 (5.0) 42 (5.2) 82 (5.1) 10 (3.5) 8 (2.8) 18 (3.2) |
| No 66 (71.0) 51 (63.8) 117 (67.6) 500 (62.0) 510 (62.6) 1010 (62.3) 185 (64.7) 189 (66.3) 374 (65.5) |
| Unknown 11 (11.8) 9 (11.3) 20 (11.6) 88 (10.9) 97 (11.9) 185 (11.4) 34 (11.9) 32 (11.2) 66 (11.6) |

Abbreviations: F-U, follow-up

^†^ Includes acute myocardial infarction, hypertension and other heart disease.

**Table 2** Per-protocol: 5-year overall mortality risk, cancer-specific mortality risk, cancer-specific recurrence risk

| Outcome | Age group | Risk in the low frequency group (95% CI) | Risk in the high frequency group (95% CI) | Risk difference |
| --- | --- | --- | --- | --- |
| Overall mortality | All | 14.5 (12.6;16.6) | 13.3 (11.5;15.3) | 1.2 (-1.6;4.0) |
| Overall mortality | ≤ 50 years | 8.6 (4.0;15.4) | 8.8 (3.8;16.3) | -0.2 (-8.6;8.3) |
| Overall mortality | 51 - 70 years | 13.3 (11.0;15.7) | 12.5 (10.4;14.9) | 0.7 (-2.5;4.0) |
| Overall mortality | > 70 years | 20.0 (15.6;24.9) | 16.9 (12.8;21.5) | 3.1 (-3.2;9.5) |
| Cancer-specific mortality | All | 11.6 (9.8;13.5) | 10.7 (9.0;12.5) | 0.9 (-1.6;3.4) |
| Cancer-specific mortality | ≤ 50 years | 7.5 (3.3;14.1) | 7.5 (3.1;14.7) | 0.01 (-7.9;7.9) |
| Cancer-specific mortality | 51 - 70 years | 11.1 (9.0;13.4) | 10.3 (8.3;12.5) | 0.8 (-2.2;3.9) |
| Cancer-specific mortality | > 70 years | 14.2 (10.4;18.6) | 12.7 (9.1;16.8) | 1.5 (-4.1;7.1) |
| Recurrence | All | 19.6 (17.4;21.9) | 21.9 (19.5;24.3) | -2.3 (-5.5;1.0) |
| Recurrence | ≤ 50 years | 21.5 (13.8;30.4) | 13.8 (7.3;22.3) | 7.8 (-3.2;18.7) |
| Recurrence | 51 - 70 years | 18.7 (16.1;21.5) | 22.7 (19.9;25.6) | -4.0 (-8.0;-0.1) |
| Recurrence | > 70 years | 21.6 (17.0;26.5) | 21.8 (17.2;26.7) | -0.2 (-7.0;6.6) |

**Table 5** Per-protocol: Cox regression analyses

| Outcome | Age group | Crude HR when comparing high-vs low-frequency groups | Adjusted HR when comparing high- vs low-frequency groups | Frailty model: Crude HR with site as a RANDOM EFFECT | Frailty model: Adjusted HR with site as a RANDOM EFFECT |
| --- | --- | --- | --- | --- | --- |
| Overall mortality | All | 0.9 (0.7;1.1) | 0.9 (0.7;1.1) | 0.9 (0.7;1.1) | 0.9 (0.7;1.1) |
| Overall mortality | ≤ 50 years | 1.0 (0.4;2.8) | 1.2 (0.4;3.7) | 1.0 (0.4;2.8) | 1.3 (0.4;3.9) |
| Overall mortality | 51 - 70 years | 0.9 (0.7;1.2) | 0.9 (0.7;1.2) | 0.9 (0.7;1.2) | 0.9 (0.7;1.2) |
| Overall mortality | > 70 years | 0.8 (0.6;1.2) | 0.8 (0.6;1.2) | 0.8 (0.6;1.2) | 0.8 (0.6;1.2) |
| Cancer-specific mortality | All | 0.9 (0.7;1.2) | 0.9 (0.7;1.1) | 0.9 (0.7;1.2) | 0.9 (0.7;1.1) |
| Cancer-specific mortality | ≤ 50 years | 1.0 (0.3;3.0) | 1.4 (0.4;4.6) | 1.0 (0.3;3.0) | 1.3 (0.4;4.6) |
| Cancer-specific mortality | 51 - 70 years | 0.9 (0.7;1.2) | 0.9 (0.7;1.2) | 0.9 (0.7;1.2) | 0.9 (0.6;1.2) |
| Cancer-specific mortality | > 70 years | 0.9 (0.6;1.4) | 0.9 (0.6;1.4) | 0.9 (0.6;1.4) | 0.9 (0.6;1.4) |
| Recurrence | All | 1.1 (1.0;1.4) | 1.1 (1.0;1.4) | 1.2 (1.0;1.4) | 1.1 (1.0;1.4) |
| Recurrence | ≤ 50 years | 0.6 (0.3;1.3) | 0.7 (0.3;1.6) | 0.6 (0.3;1.3) | 0.7 (0.3;1.6) |
| Recurrence | 51 - 70 years | 1.3 (1.0;1.6) | 1.3 (1.0;1.6) | 1.3 (1.0;1.6) | 1.3 (1.0;1.6) |
| Recurrence | > 70 years | 1.0 (0.7;1.5) | 1.0 (0.7;1.4) | 1.0 (0.7;1.5) | 1.0 (0.7;1.4) |

**Fig. 10** Per-protocol: 5-year overall mortality rate for all age-groups


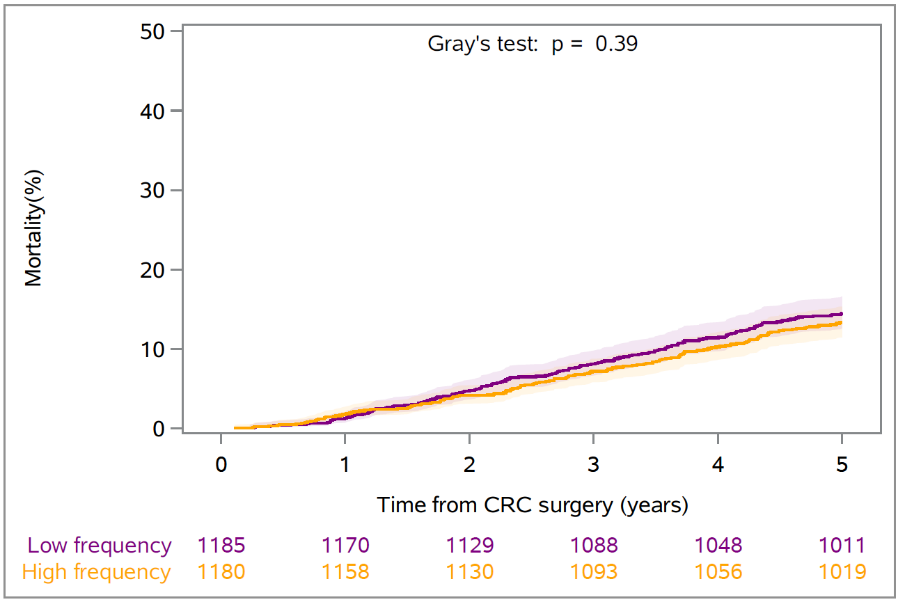


**Fig. 11** Per-protocol: cancer-specific mortality rate for all age-groups


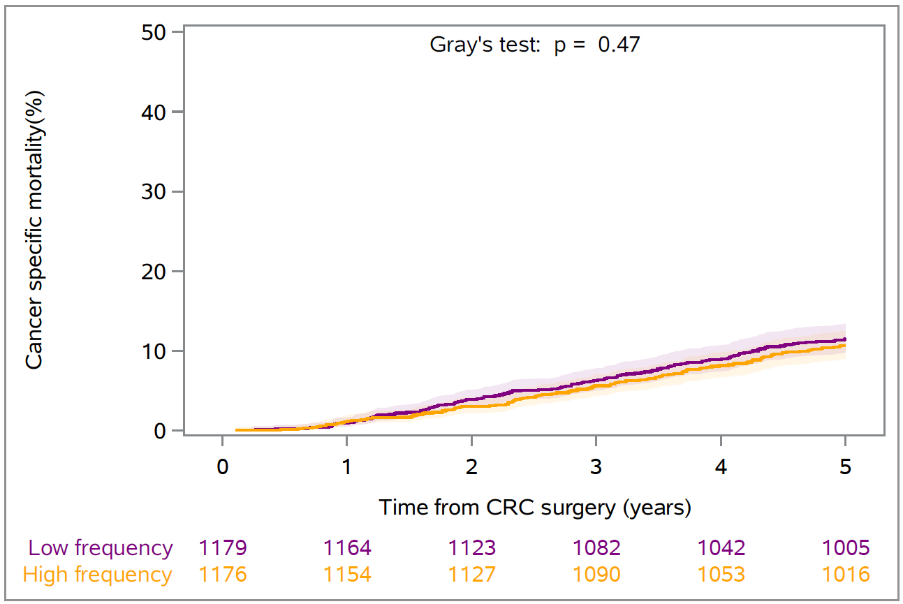


**Fig. 12** Per-protocol: cancer-specific recurrence rate for all age-groups


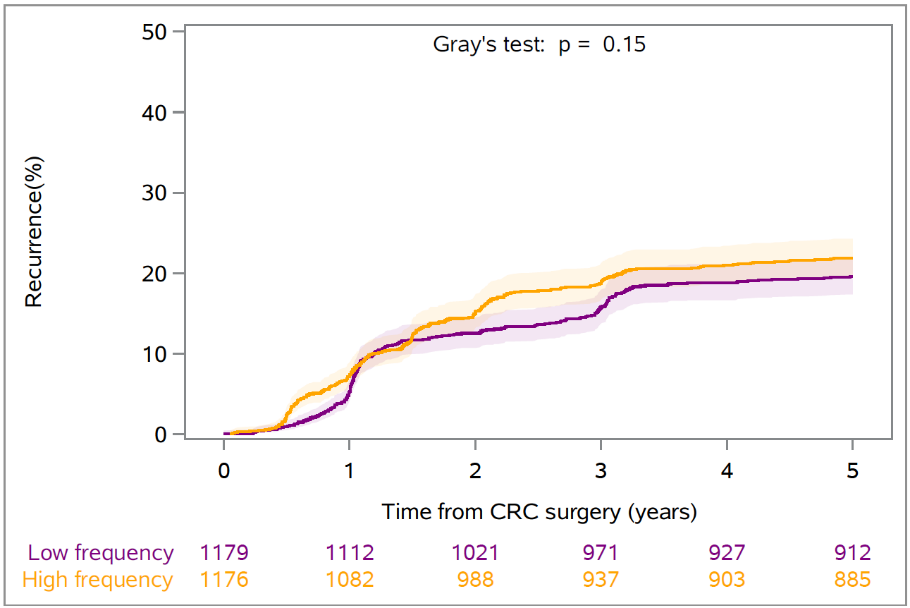


**Fig. 13** Per-protocol: 5-year overall mortality rate for patients ≤ 50 years


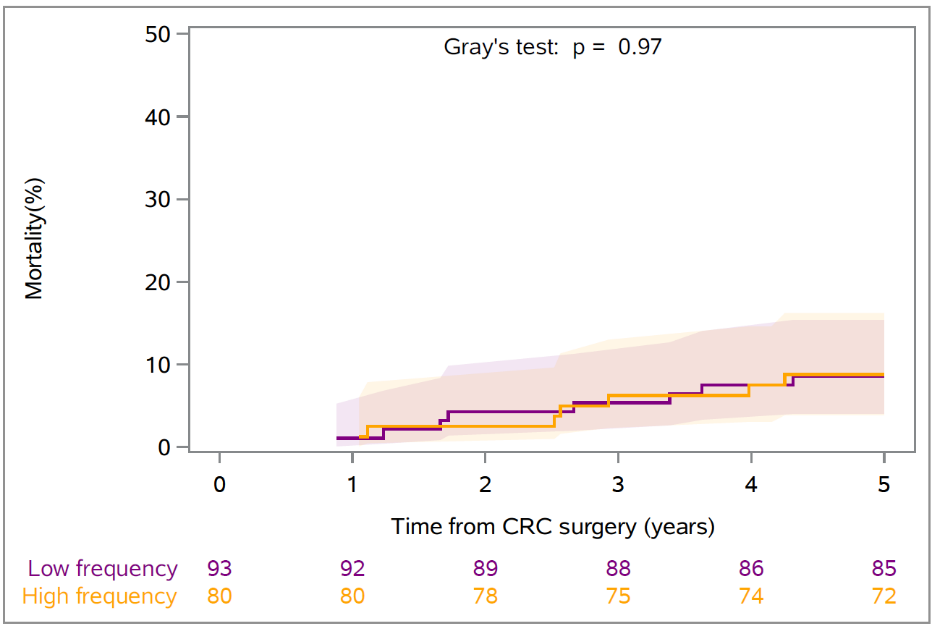


**Fig. 14** Per-protocol: 5-year overall mortality rate for patients 51-70 years


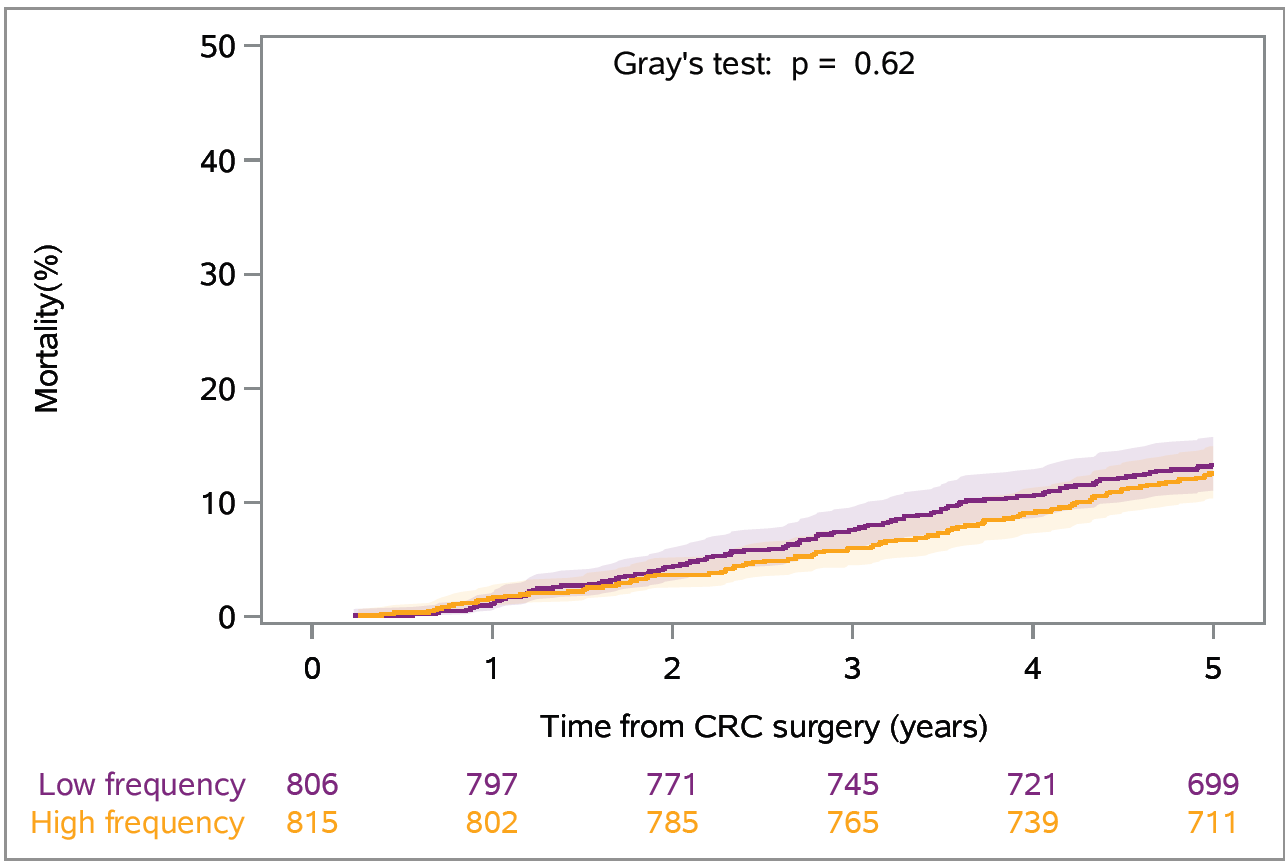


**Fig. 15** Per-protocol: 5-year overall mortality rate for patients > 70 years


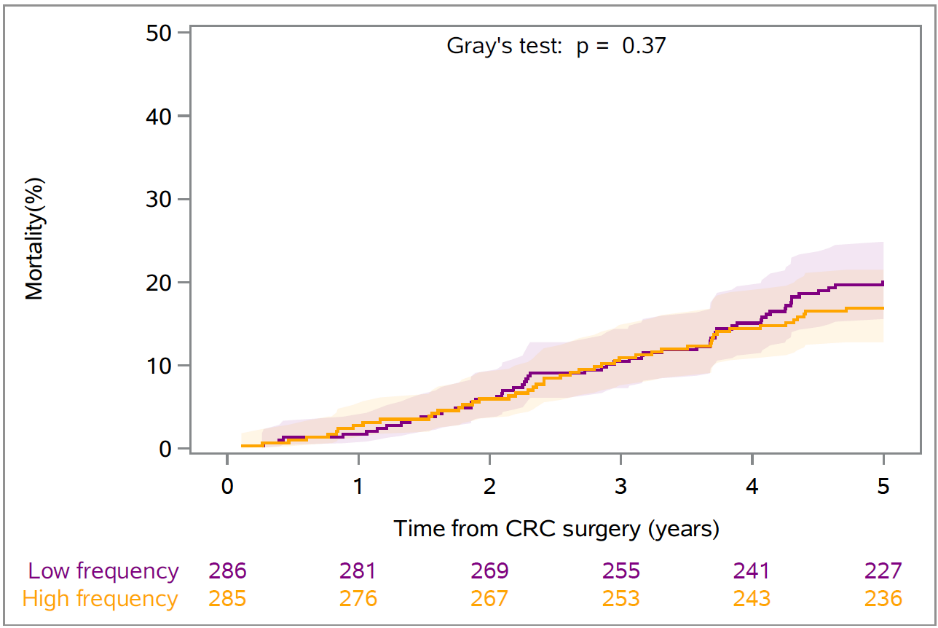


**Fig. 16** Per-protocol: cancer-specific mortality rate for patients ≤ 50 years


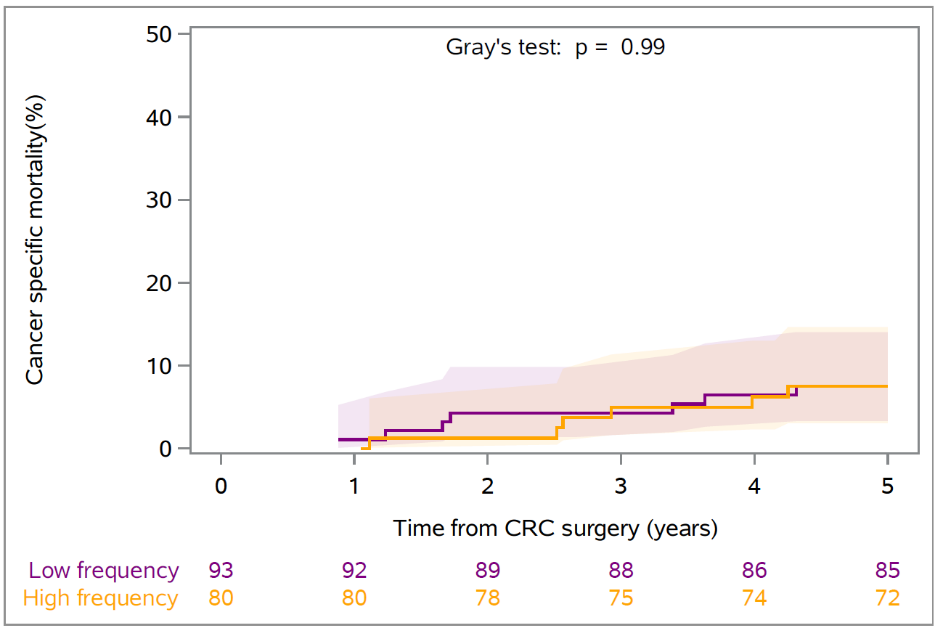


**Fig. 17** Per-protocol: cancer-specific mortality rate for patients 51-70 years


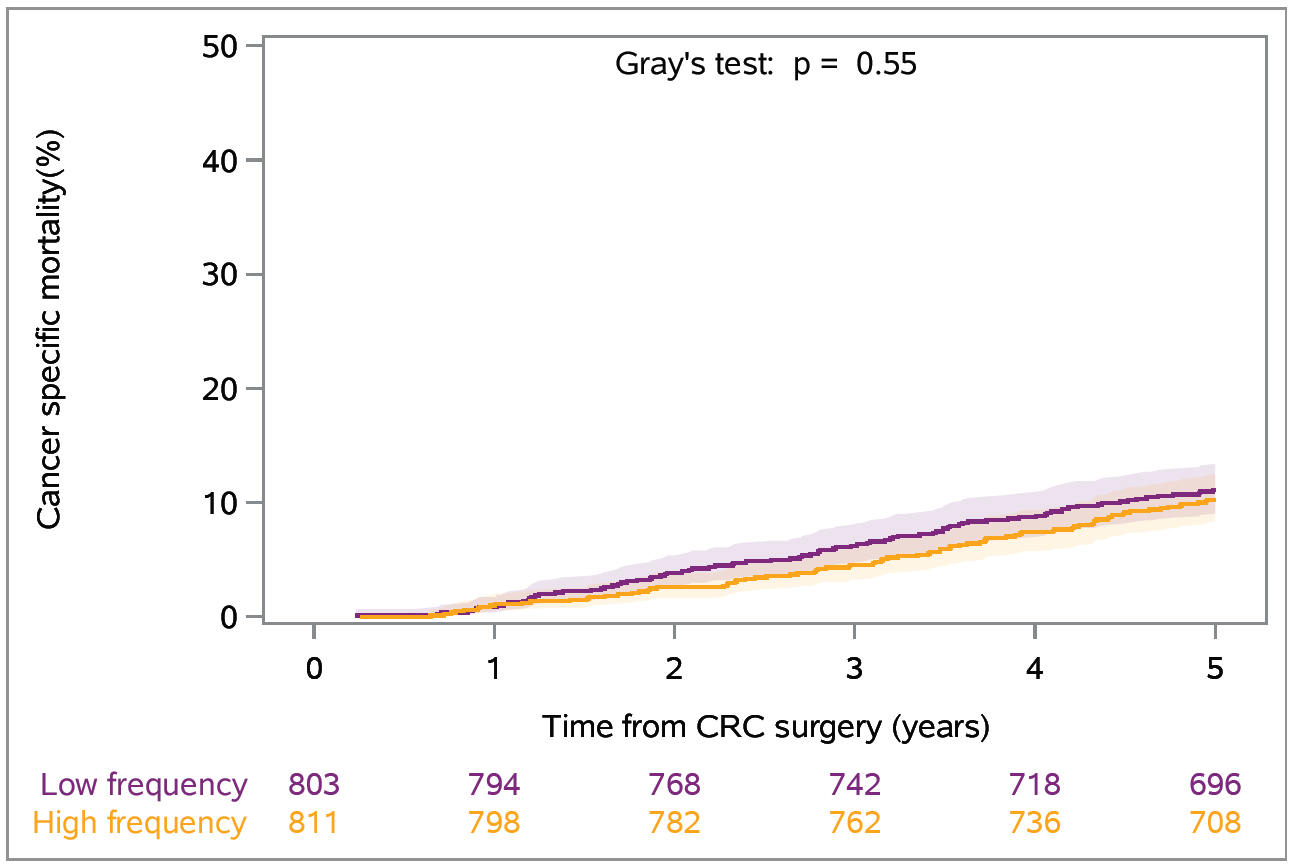


**Fig. 18** Per-protocol: cancer-specific mortality rate for patients > 70 years


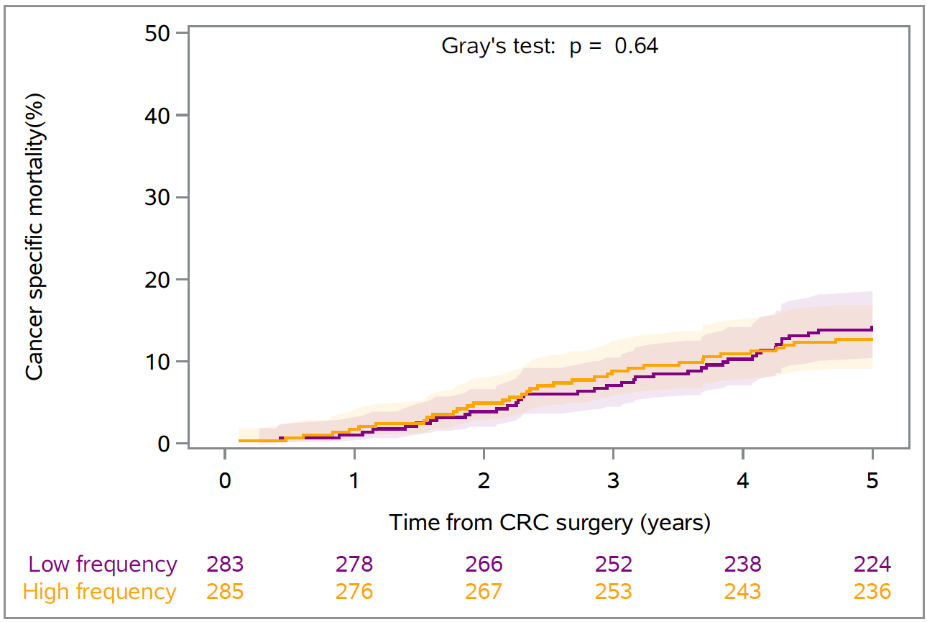


**Fig. 19** Per-protocol: cancer-specific recurrence rate for patients ≤ 50 years


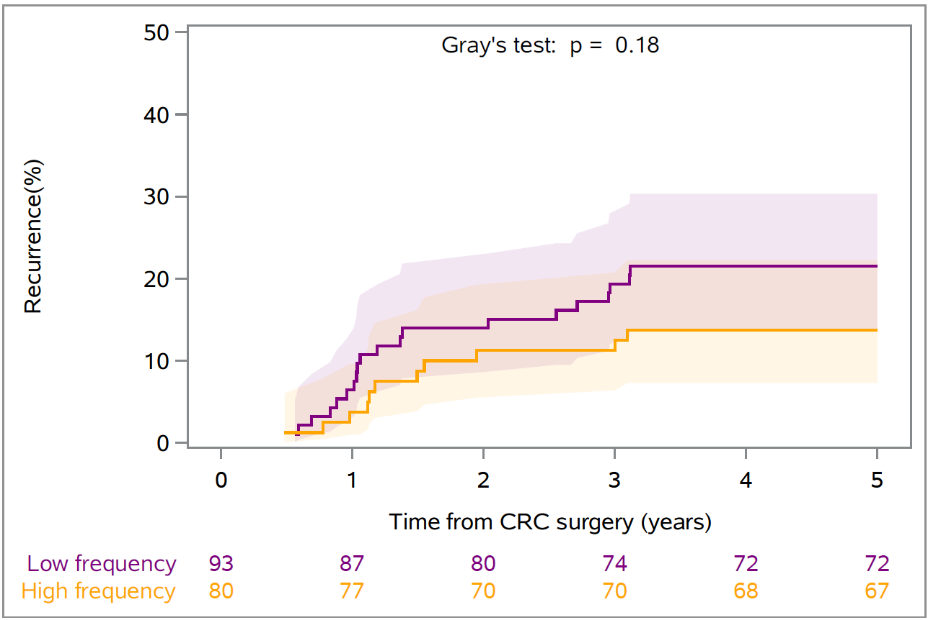


**Fig. 20** Per-protocol: cancer-specific recurrence rate for patients 51-70 years


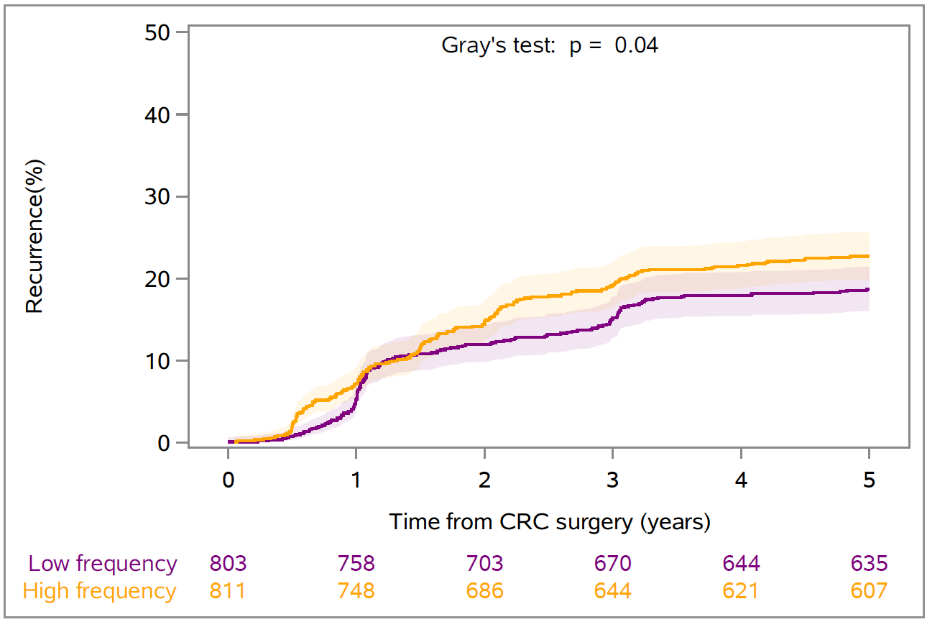


**Fig. 21** Per-protocol: cancer-specific recurrence rate for patients > 70 years


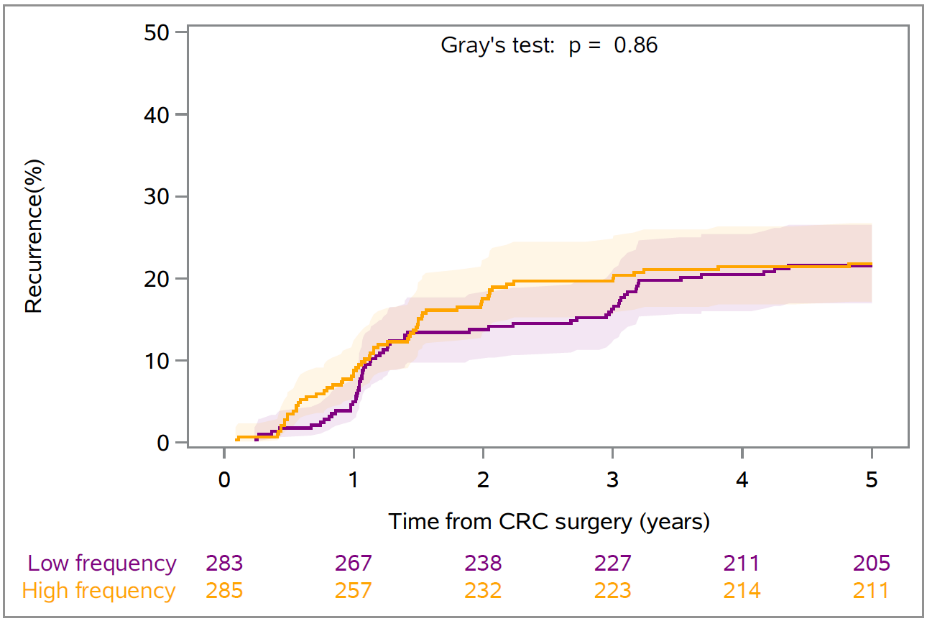

Supplement: Supplementary file 1 — DOCX (1.24 MB) [file 384_2026_5096_MOESM1_ESM.docx]
